# Supplementary material for: Co-coding of head and whisker movements by both VPM and POm thalamic neurons
Source: Nat Commun. 2024 Jul 13;15:5883. doi: 10.1038/s41467-024-50039-z (PMC11246487; doi:10.1038/s41467-024-50039-z)
Supplement: Supplementary file 1 — Supplementary Information [file 41467_2024_50039_MOESM1_ESM.pdf]

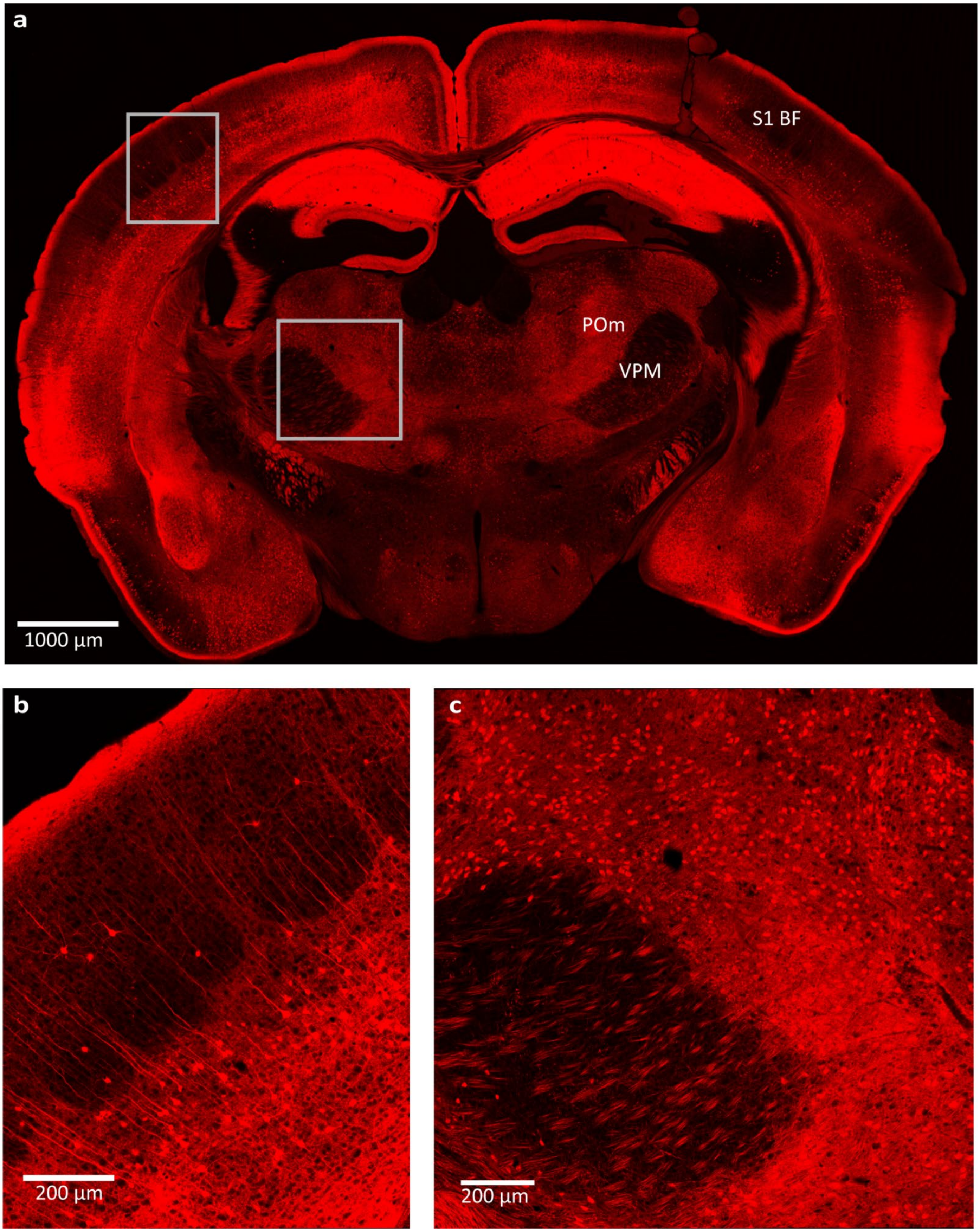

Supplementary Figure 1: **Expression of tdTomato in GPR26-Cre X Ai9 mice. a,** The GPR26-Cre mouse line expresses Cre densely in the POm and only sparsely in the VPM, as evident in tdTomato-positive cells in both nuclei. **b,** Axonal projections can be seen in the barrel septa, layer 5a and layer 1, but few projections can be seen in the layer 4 barrels of the primary somatosensory cortex barrel field (S1BF). Boxed area in (a) is enlarged in (b). **c,** Enlarged image of the thalamus from boxed area in (a).

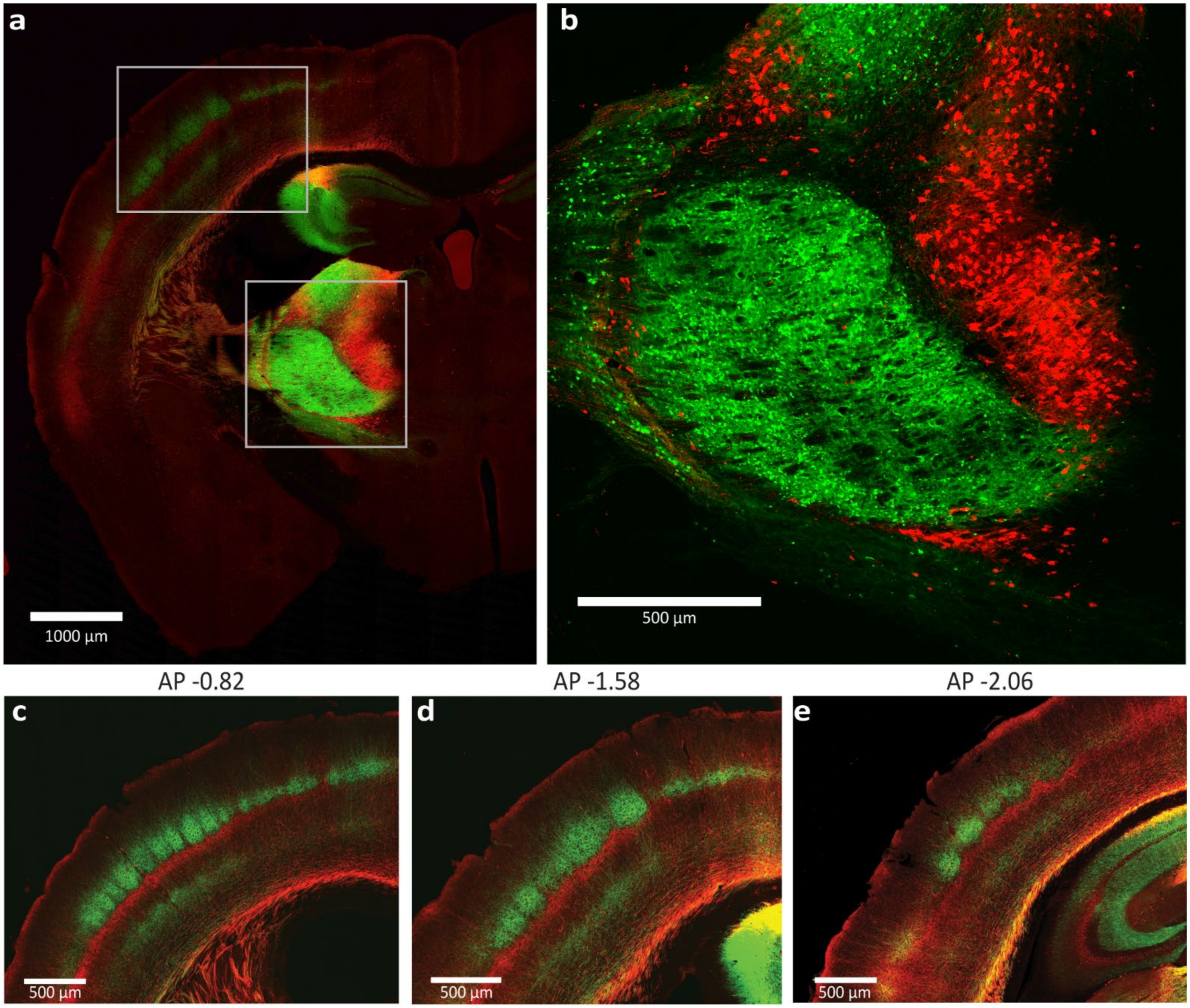

Supplementary Figure 2: **Expression of Cre-ON and Cre-OFF viral constructs. a,** The Cre-ON/Cre-OFF genetic targeting strategy can be used to simultaneously and differentially target the expression of EYFP to the VPM (green) and mCherry to the POm (red). **b,** Enlarged image of the thalamus from boxed area in (a). **c-e,** Pathway specific thalamocortical projections of EYFP-expressing VPM neurons (green) and mCherry expressing POm neurons (red) can be seen throughout the barrel cortex. Boxed area in (a) is enlarged in (d).

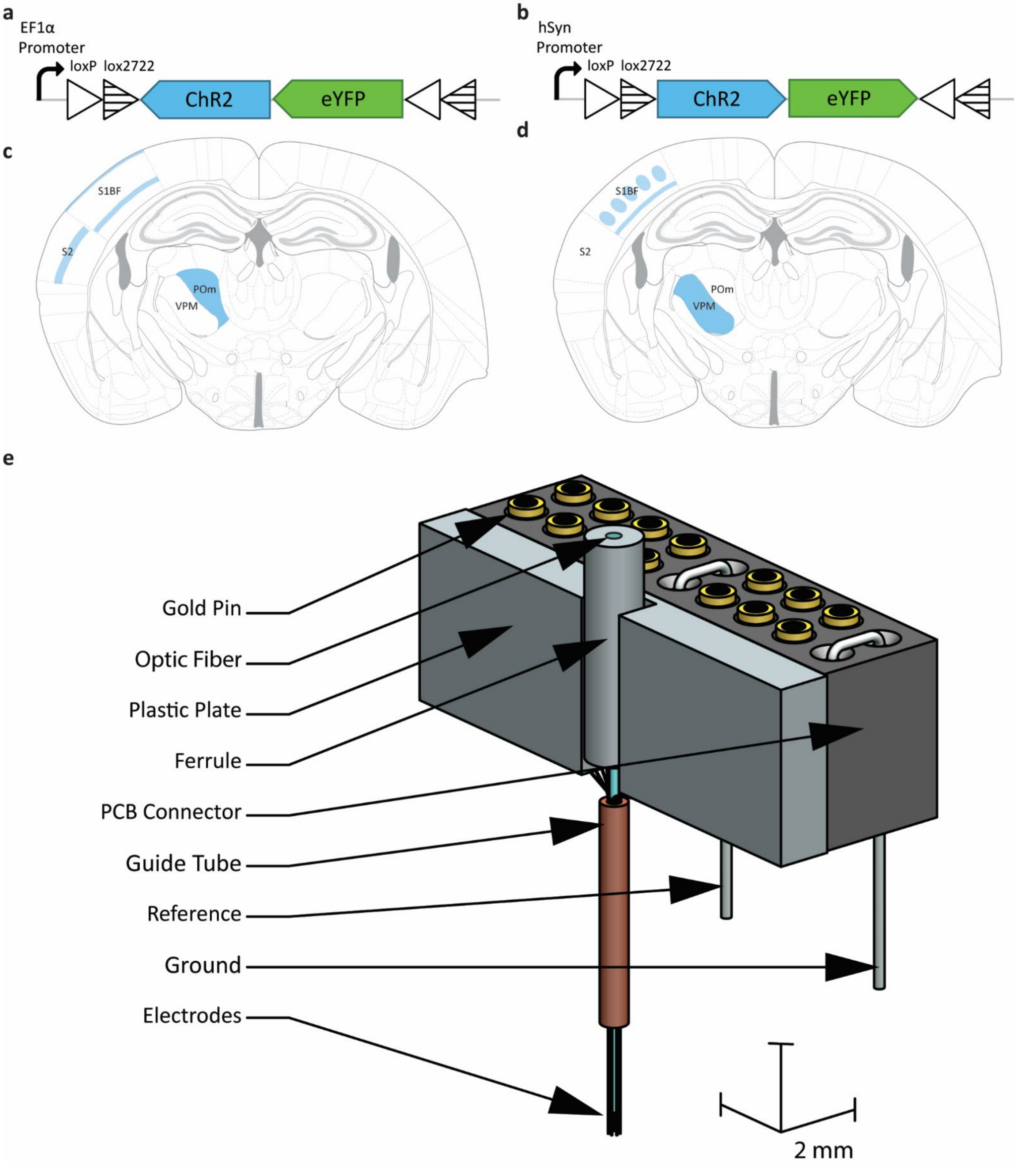

Supplementary Figure 3: **DFO/DIO viral constructs used in behavioral experiments and chronic multi-site optrodes. a,** Schematic of the Cre-ON viral construct that was unilaterally injected to the whisking related nuclei of the thalamus to target expression of ChR2-EYFP to the POm. **b,** Schematic of the Cre-OFF viral construct that was unilaterally injected to the whisking related nuclei of the thalamus to target expression of ChR2-EYFP in the VPM. **c,** Expected expression pattern of Cre-ON viral construct. **d,** Expected expression pattern of Cre-OFF viral construct. Images in c and d were modified with permission from Paxinos, G. and K. B. Franklin (2004) *The mouse brain in stereotaxic coordinates*, Gulf Professional Publishing. **e,** Implanted chronic multi-site optrodes (CMOs) were used to record the neuronal activity of awake, behaving mice while delivering laser stimulation.

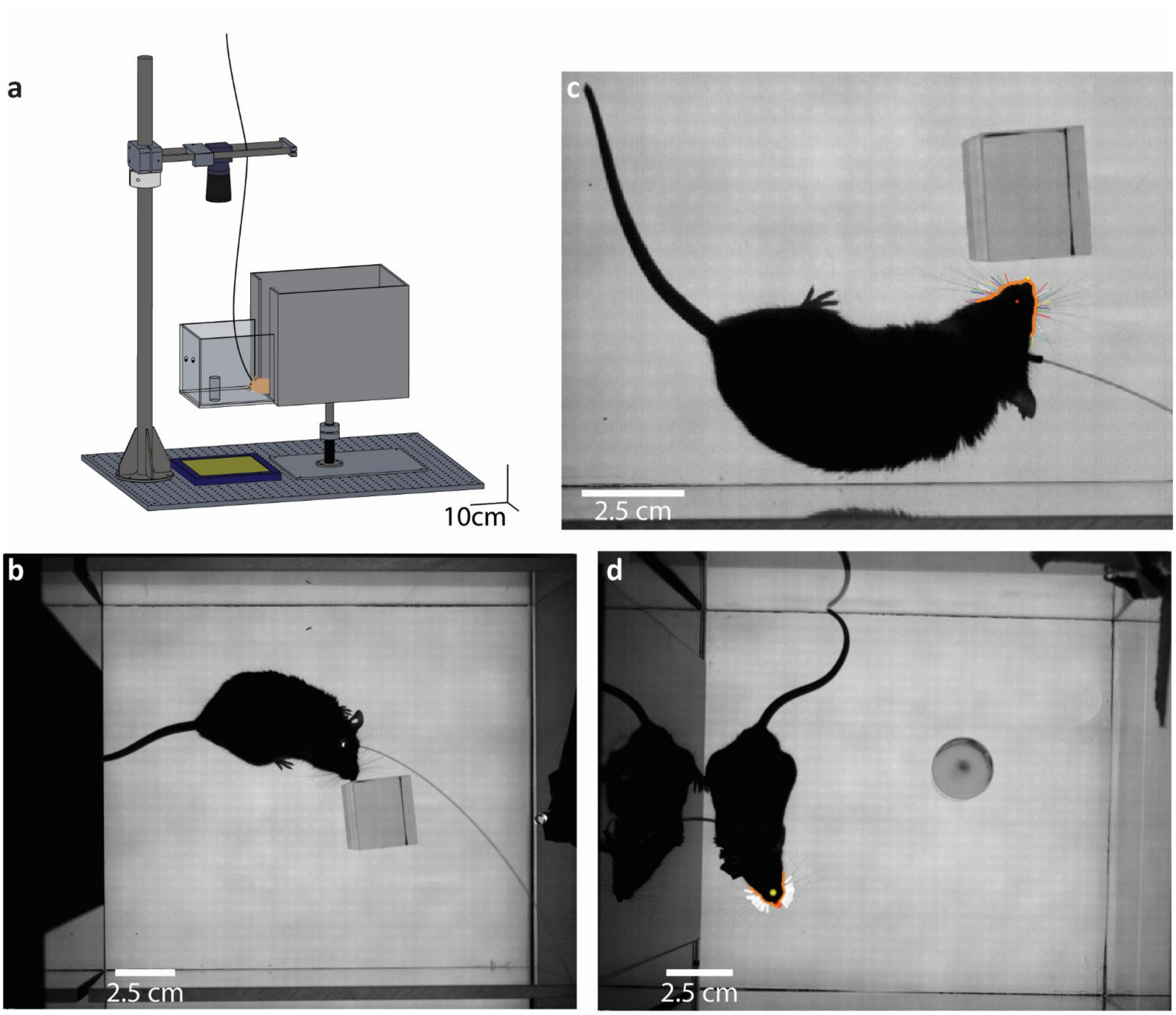

Supplementary Figure 4: **OptoWhisk behavioral apparatus. a,** Mice performed behavioral assays in the custom-designed OptoWhisk. **b,** Example video frame. An IR-backlight is placed beneath the behavioral arena. A high-speed, high-resolution camera enables the visualization of fine whiskers. **c and d,** Head-motion and whiskers were tracked offline using the BIOTACT Whisker Tracker (BWT).

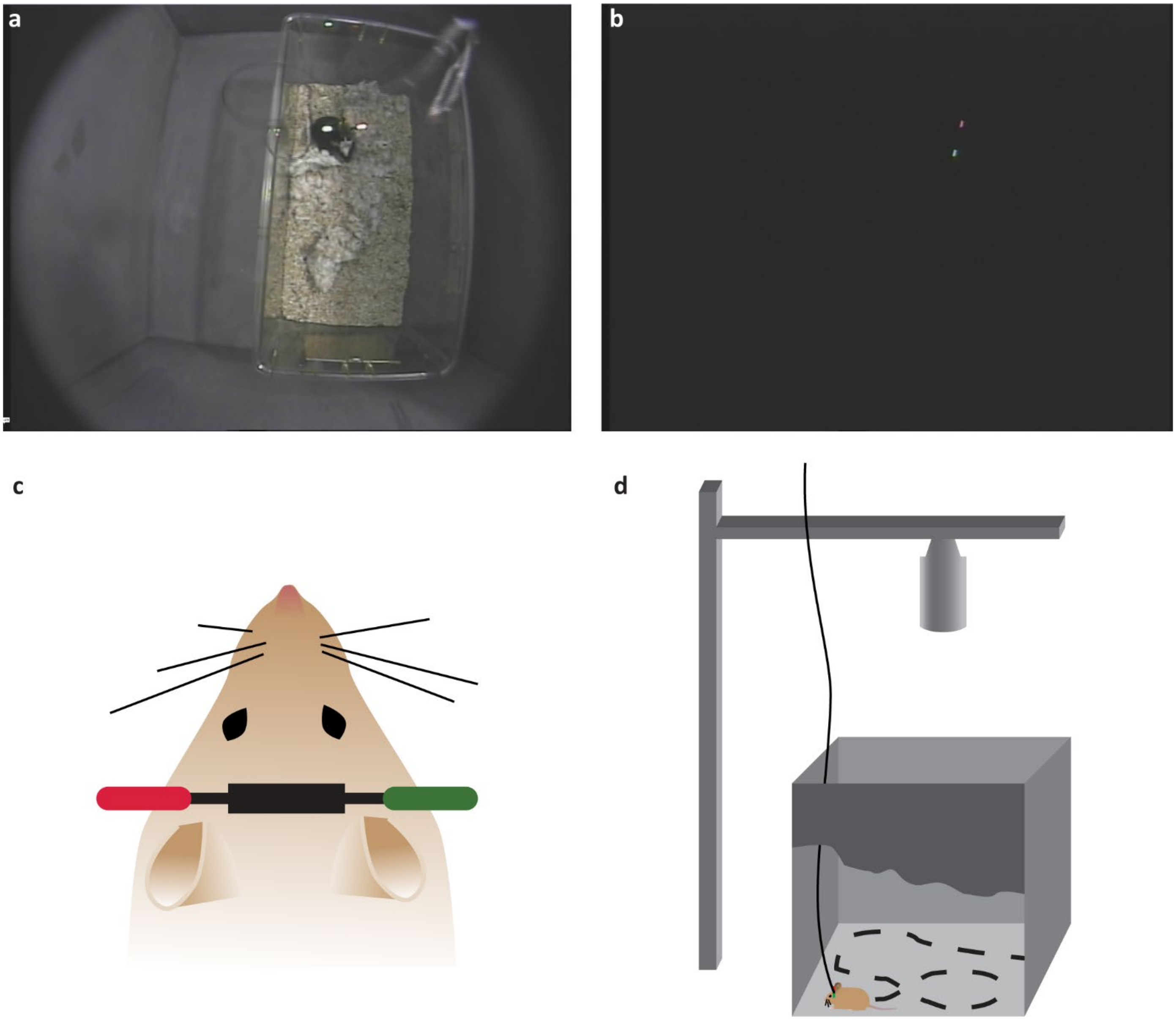

Supplementary Figure 5: **Open Field behavioral apparatus. a,** The photo-tagging portion of the open field sessions was performed while the mouse was in its home cage. The mouse's home cage was placed within the open field box. Lights are on in this example frame for illustrative purposes.**b,** During the open field behavioral assay, the mouse was moved from its home cage into the open field box. Both the photo-tagging and behavioral portions of the experiment were performed in the dark. **c,** The position of the mouse was automatically tracked using red and green LEDs placed on either side of the headstage. **d,** The freely-moving mice were filmed from above.

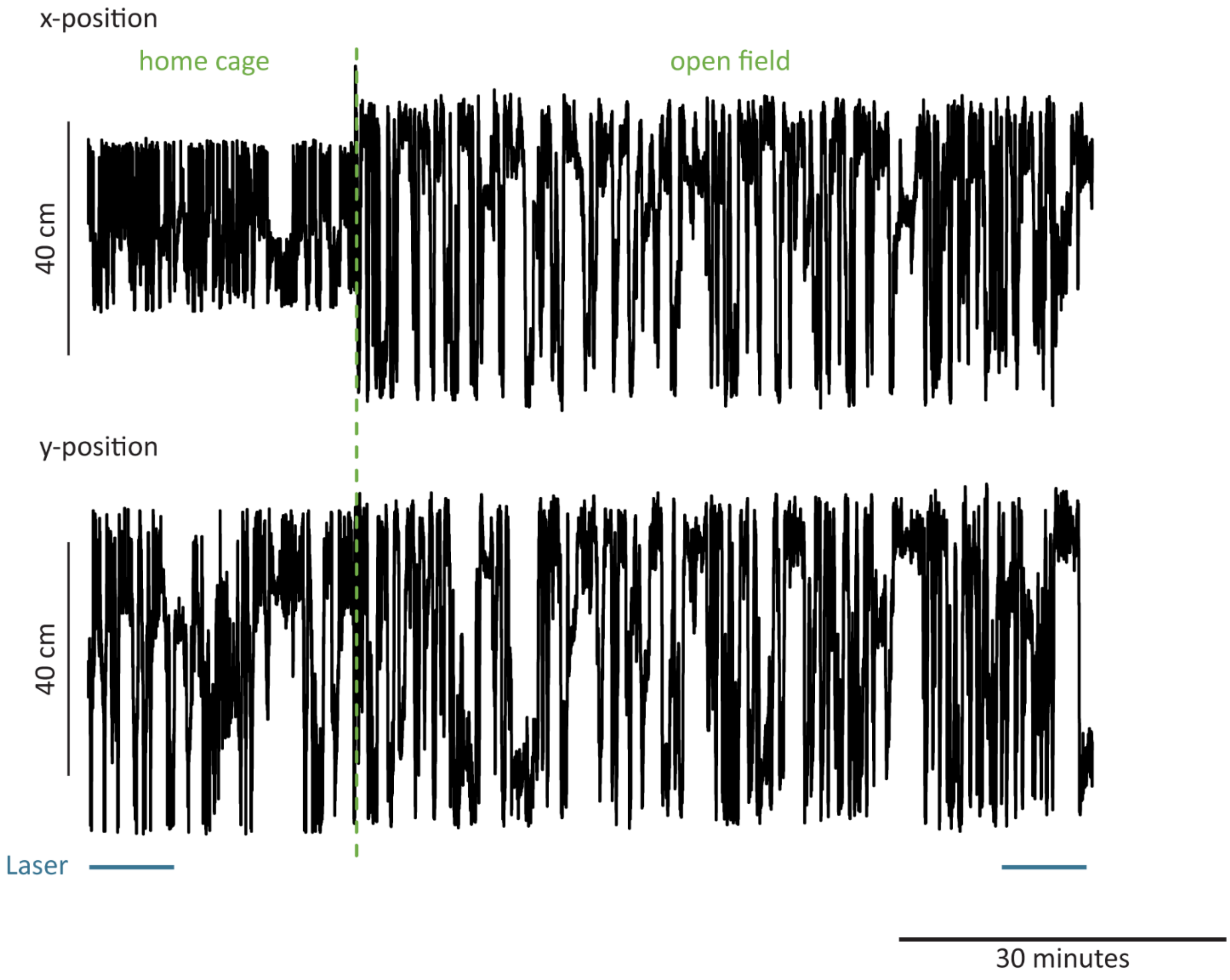

Supplementary Figure 6: **Example trial in Open Field session.** X- and y-position of a mouse expressing ChR2 in the POm during an example open field session. Periods of laser stimulation of POm units are indicated in blue. Dashed green vertical line indicates when the mouse was transferred from its home cage into the open field box.

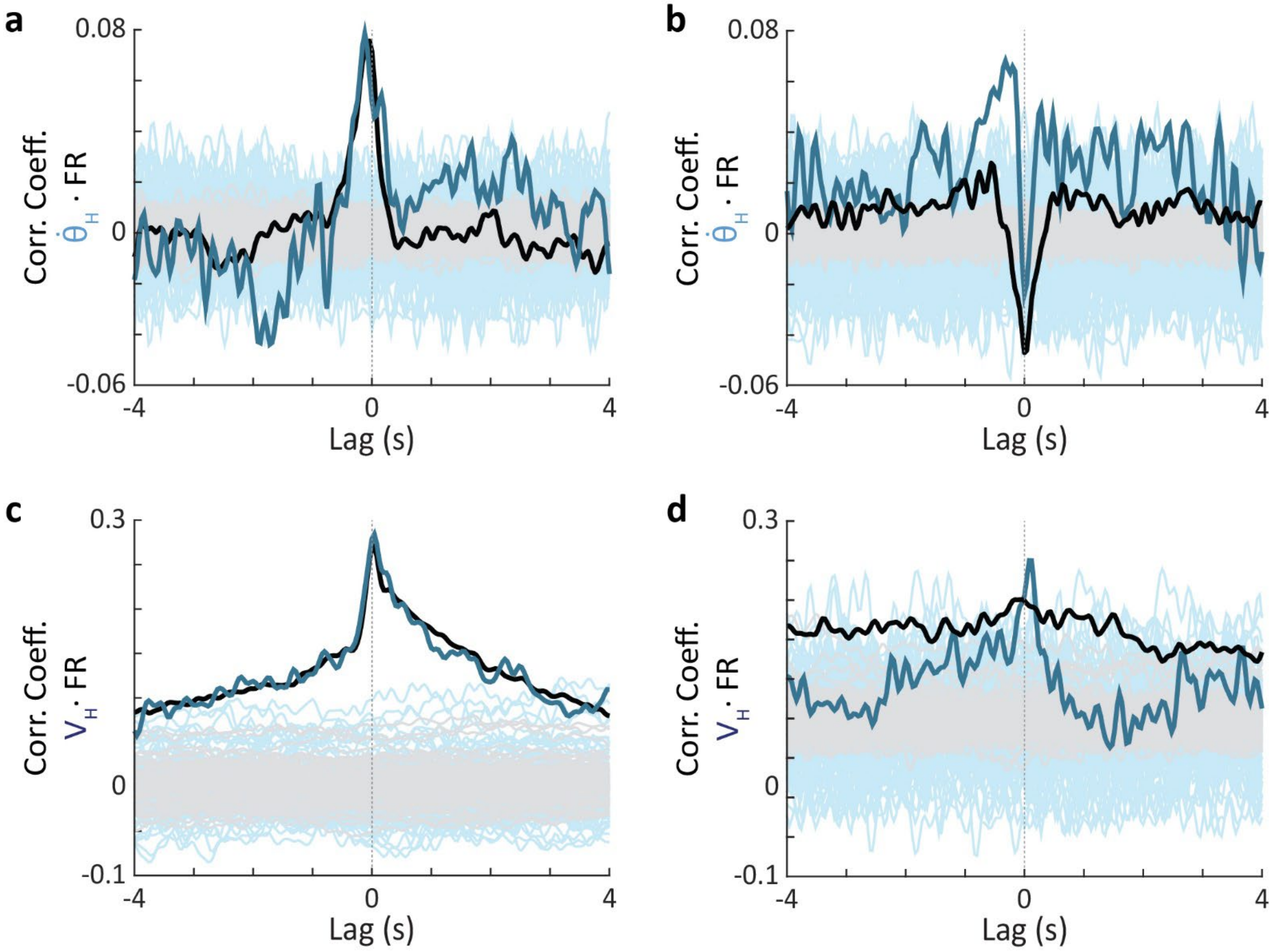

Supplementary Figure 7: **Cross-correlations between neuronal populations and head kinematics.** Cross-correlation functions between neuronal firing-rate functions (FR) and kinematic variables ($\dot{\theta}_H$ in panels a, b and V_H in panels c, d) for VPM (a, c) and POm (b, d) neuronal populations, during free behavior (black) or laser stimulation (bluish). Low-intensity curves are cross-correlations between shuffled traces (100 repetitions).

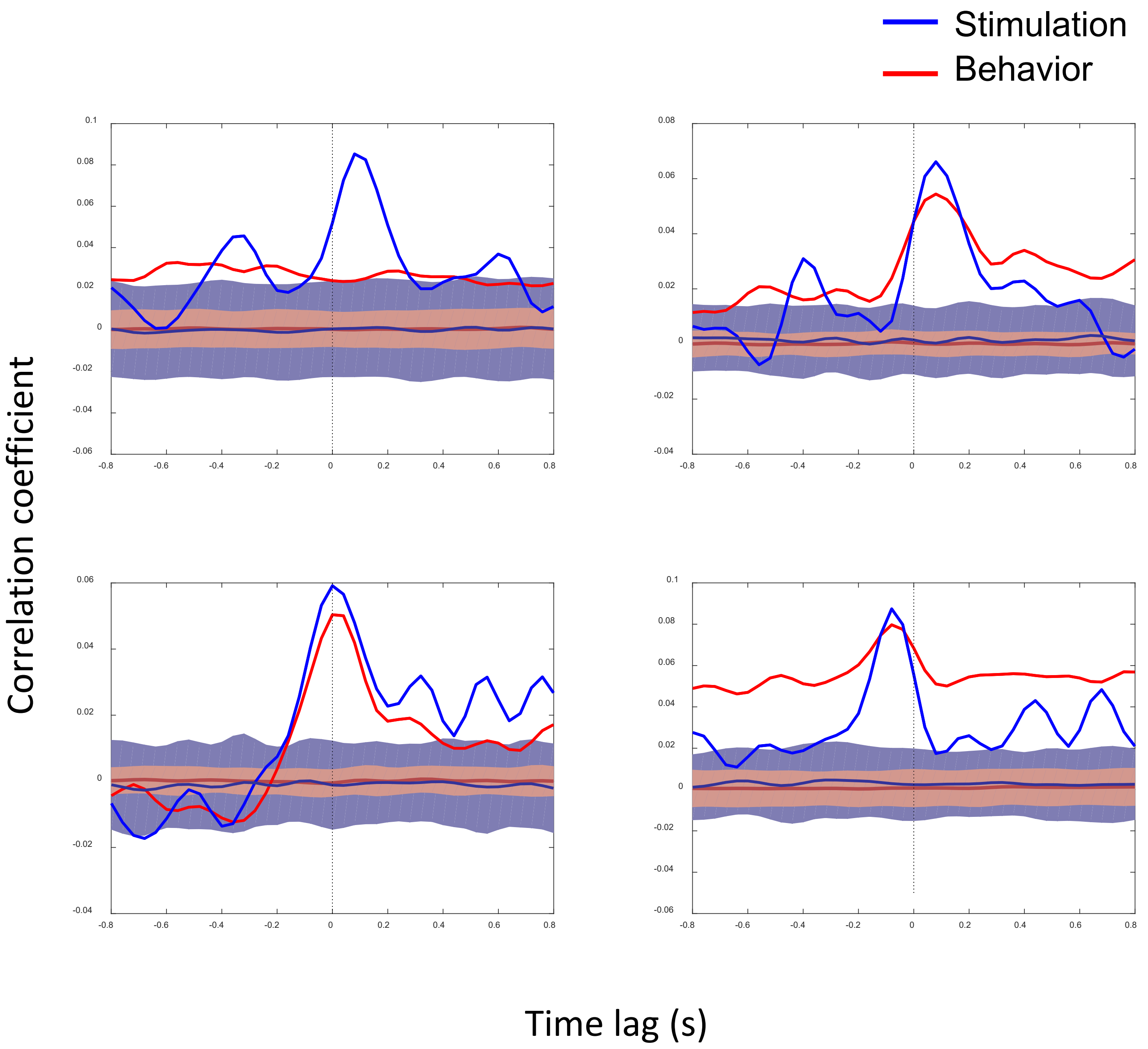

Supplementary Figure 8: **Cross-correlations between single-units and head linear velocity.** Cross-correlation functions between spikes of four individual thalamic neurons and head linear velocity during free behavior (red) or laser stimulation (blue). Cross-correlation functions of shuffled data (100 repetitions) are depicted by their means (dark) and 1 SD (light bands,) in red and blue, respectively. Cases where the entire empirical curve is above the shuffled mean are cases with broad correlation peaks (e.g., Supplementary Fig. 7).

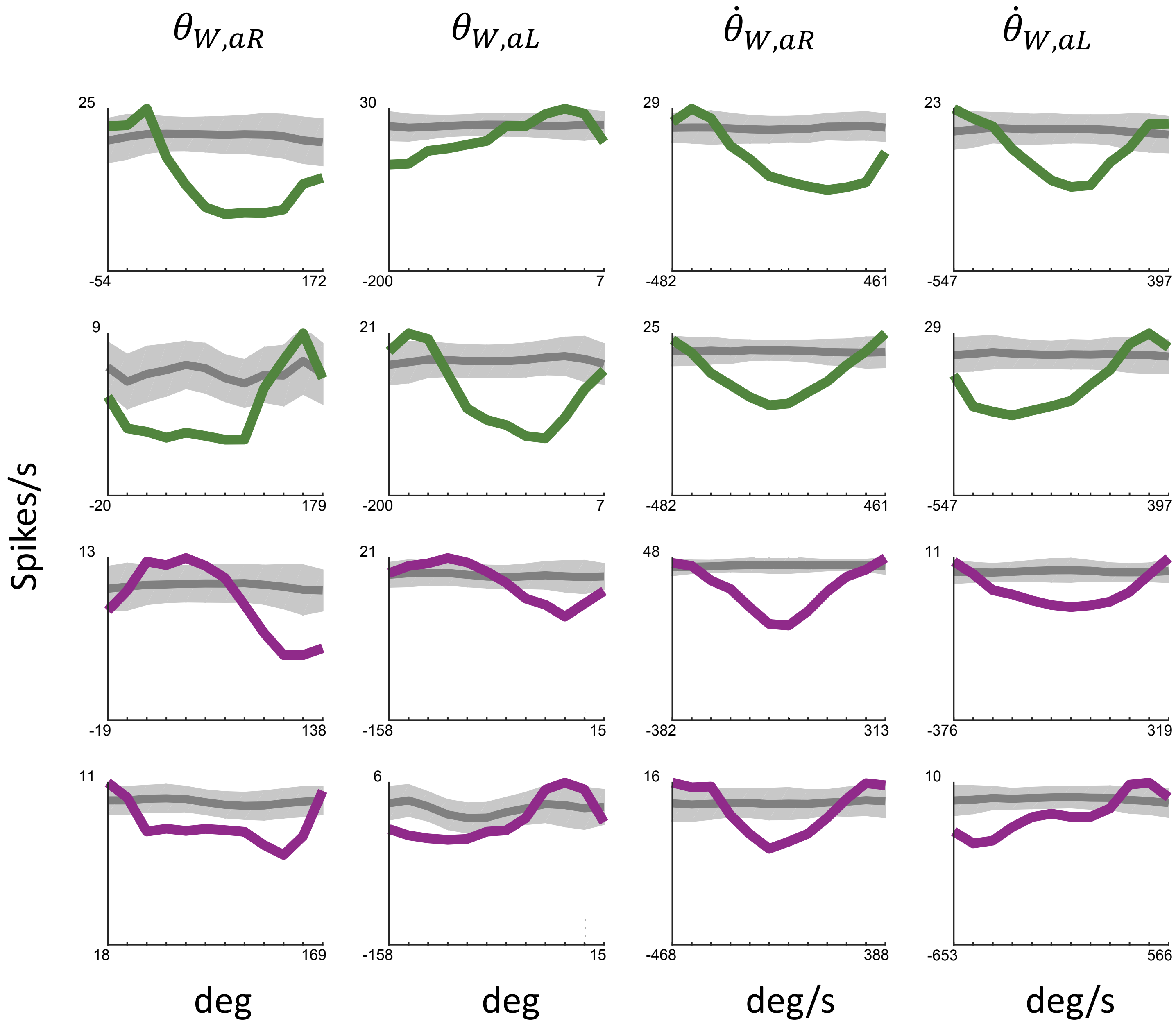

Supplementary Figure 9: **Allocentric tuning curves.** Examples of single-unit tuning curves for allocentric (relative to the external frame, see Methods) variables. $\theta_{W,aR}$ and $\theta_{W,aL}$are allocentric whisker angles of right and left whisker beams, respectively, and $\dot{\theta}_{W,aR}$ and $\dot{\theta}_{W,aL}$ are allocentric whisker velocities. Tuning curves of shuffled data are depicted by their mean (dark gray) and 1 SD (light gray). Purple, POm; Green, VPM. Each curve is normalized to its maximal value. The firing rates labeling the ordinate in each plot refer to the empirical data only.
