# Supplementary material for: Co-coding of head and whisker movements by both VPM and POm thalamic neurons
Source: Nat Commun. 2024 Jul 13;15:5883. doi: 10.1038/s41467-024-50039-z (PMC11246487; doi:10.1038/s41467-024-50039-z)
Supplement: Supplementary file 3 — Description of Additional Supplementary Files [file 41467_2024_50039_MOESM3_ESM.pdf]

Description of Additional Supplementary Files

Supplementary Movie 1 - An example of a spontaneous emergence task. Video slowed down x16.7

Supplementary Movie 2 - An example of the results of laser stimulations. A single block (12 repetitions) of laser stimulations at 2 Hz in the VPM (Fig. 4a), followed by 4 repetitions of 5 Hz stimulations, are shown. Audio conveys neuronal activity. Stimulation times are denoted by a blue rectangle at the top-right corner. The red and green LEDs were placed on the left and right sides of the recording headstage, respectively.
